# Computational classification of MocR transcriptional regulators into subgroups as a support for experimental and functional characterization

**DOI:** 10.6026/97320630015151

**Published:** 2019-02-28

**Authors:** Stefano Pascarella

**Affiliations:** 1 Structural bioinformatics and Molecular modelling Lab;Dipartimento di Scienze biochimiche;Sapienza Universita di Roma;00185 Roma,Italy

**Keywords:** MocR, Pyridoxal 5'-phosphate, Structural bioinformatics, Aspartate aminotransferase, Multidimensional scaling analysis, Cluster analysis

## Abstract

MocR bacterial transcriptional regulators are a subfamily within the GntR family. The MocR proteins possess an N-terminal domain containing the winged Helix-Turn-Helix (wHTH) motif and a C-terminal domain whose architecture is homologous to the fold type-I pyridoxal 5'-phosphate (PLP) dependent enzymes and whose archetypical protein is aspartate aminotransferase (AAT). The ancestor of the fold type-I PLP dependent super-family is considered one of the earliest enzymes. The members of this super-family are the product of evolution which resulted in a diversified protein population able to catalyze a set of reactions on substrates often containing amino groups. The MocR regulators are activators or repressors of gene control within many metabolic pathways often involving PLP enzymes. This diversity implies that MocR specifically responds to different classes of effector molecules. Therefore, it is of interest to compare the AAT domains of MocR from six bacteria phyla. Multi dimensional scaling and cluster analyses suggested that at least three subgroups exist within the population that reflects functional specialization rather than taxonomic origin. The AAT-domains of the three clusters display variable degree of similarity to different fold type-I PLP enzyme families. The results support the hypothesis that independent fusion events generated at least three different MocR subgroups.

## Background

The vast GntR family [1,2] was named after GntR, a regulator found to be involved in the expression of the gluconate operon of Escherichia coli K12 [3] and Bacillus subtilis [4]. Since then, GntR transcriptional factors have been found widely distributed in eubacteria and involved in the regulation of various, important biological processes [5,6]. The proteins belonging to this family possess a characteristic molecular architecture, which includes a Nterminal DNA-binding domain containing the well-known winged Helix-Turn-Helix (wHTH) motif [7] and a C-terminal domain with oligomerization and/or effector binding function [8,9]. The two domains are interconnected through a peptide linker of variable length in different GntRs [10,11]. The C-terminal domain of GntRs can belong to different protein families, which attribute functional and effector specificity diversification to the transcriptional regulator. To date, seven different subfamilies have been observed for the C-terminal domain [5]. Among these, the MocR subfamily was denominated after the GntR regulator of the moc genes involved in the 3-O-MSI (L-3-O-methyl-scyllo-inosamine) catabolism discovered in Rhizobium melitoti [12,13]. This subfamily is characterised by a large C-terminal domain, whose protein architecture is homologous to the fold type-I pyridoxal 5'- phosphate (PLP) dependent enzymes [14,15]. Aspartate aminotransferase (AAT) epitomizes fold type-I PLP-dependent enzymes. The PLP cofactor is covalently bound via its aldehyde group to an active site lysine residue forming a Schiff base, while the phosphate group is anchored to the enzyme via hydrogen bonds and salt bridges. These enzymes frequently exist as homodimers in which the active site pocket is located in proximity of the subunit interface [16]. The ancestor of the fold type-I PLP dependent enzyme superfamily is considered one of the earliest enzyme appeared on Earth [15,17]. Consequently, the members of this superfamily must be the product of a long and intricate evolution which led up to a much diversified superfamily able to exploit the PLP chemistry to catalyse reactions on specific substrates, generally containing amino groups [18]. Consequently, type-I PLP dependent enzymes are considered one of the top most five "polymath" enzyme super families [19]. Since their discovery, several MocR regulators have been studied and characterized: TauR activates the expression of taurine utilization genes in Rhodobacter capsulatus [20]; Bacillus subtilis GabR with PLP and γ- aminobutyric acid (GABA) bound as external aldimine, activates transcription of genes coding for GABA aminotransferase and succinic semi aldehyde dehydrogenase [21]; PtsJ regulates the production of pyridoxal kinase in Salmonella typhimurium [22] while PdxR is involved in the regulation of the PLP synthesis in several bacteria such as Bacillus clausii [23]. More examples are reported in a recently published review [2].

In general, the MocR regulators are involved as activators or repressors in the control of many, important metabolic networks not yet fully characterized but often involving PLP-dependent enzymes [6,24]. Moreover, subgroups of MocR were predicted to regulate genes coding for different types of proteins including membrane transporters [25,26]. Despite their relevance, very little is known about the molecular mechanism underlying their function, their response to effector binding, and the molecular structure of the effectors. To date, only the crystallographic structure of B. Subtilis GabR is available in the Protein Data Bank (PDB) [27,28]. More recently, the structures of the dimeric GabR AAT domains in complex with the external aldimine formed by the PLP and the GABA have been deposited by two different research groups [29,30]. Molecular dynamics simulations [31] have underlined the flexibility of the linker. Experiments of atomic force microscopy have suggested as well that effector binding triggers a conformational modification in GabR [32].

MocRs represent an interesting case of evolution of chimeric proteins [33]. To verify whether subgroups can be discovered within the whole MocR population, a comprehensive comparison has been carried out among the AAT domains of regulator sequences from six bacteria phyla. Cluster analysis techniques suggested that the AAT-domain sequences fall into three subgroups of heterogeneous taxonomic composition. Each subgroup displays a different degree of similarity to respective families of fold type-I PLP dependent enzymes. It may be speculated that independent fusion/recombination events between wHTH domains and catalytically specialized PLP-dependent enzymes of fold type-I generated at least three different MocR subgroups, each of which characterized by specificity for a class of effector molecules, originated from the parent enzyme.

## Methodology

### Data set collection and sequence alignment:

Bacterial protein sequences from complete proteomes have been retrieved for each phylum from UniProt Data Bank [34] accessed on October 2017. MocR sequences have been extracted from the collected UniProt sets using the program rps-blast [35]: only sequences containing both a N-terminal HTH domain and a Cterminal AAT-like domain were considered genuine MocR proteins. Sequences have been split into the HTH and AAT domains. Domain boundaries have been determined by alignment of each MocR sequence to the HTH and AAT profile definitions available in the CDD databank [36]. To reduce redundancy, sequences were filtered with the cd-hit software tool [37] so that no pair of the remaining sequences shared more than 50% sequence identity. Multiple sequence alignments were calculated using the program clustal omega [38]. Jalview [39] was the editor utilized to align and analyse sequences. PyMOL [40] and Chimera [41] were the molecular graphics tools. Bash, Perl and R scripts within Rstudio environment [42] were used for file processing and data analysis.

### Multidimensional scaling and cluster analysis:

Multidimensional scaling (MDS) and clustering techniques as implemented in R modules "bios2mds" [43] and "cluster" have been applied. The package "bios2mds" provides functions to analyse multiple sequence alignments of homologous proteins. The multiple alignments can be converted into a distance matrix containing the pairwise differences calculated using a scoring matrix such as BLOSUM30. MDS analysis can assign to each sequence represented in the distance matrix a set of coordinates in the principal component space. K-means cluster analysis can inspect the distribution of sequences in the projection space and subdivide them into a predetermined number of clusters.

### Hidden Markov Model (HMM) profile searches:

HMMer suite [44] was employed to correlate the MocR AAT domain clusters to existing families of fold type-I PLP-dependent enzymes. Sequences attributed to each cluster by K-means analysis have been multiply aligned. Each alignment has been converted into a HMM profile that has been searched over the Pfam-A domain databank [45] and output parsed by bash scripts.

### Logo comparisons and structure analysis:

Differences discriminating the sequence clusters have been tentatively identified through Seq2Logo web server (http://www.cbs.dtu.dk/biotools/Seq2Logo/). Seq2Logo [46] displays a graphical representations of the residue frequency within each column of a multiple sequence alignment. Logos calculated for each cluster have been compared and the observed differences have been mapped onto the GabR structure with PDB ID 5x03.

## Results

### Data collection and processing:

The proteomes of bacteria from the most populated phyla were considered to the purpose of this analysis: Actinobacteria, Firmicutes, Alpha proteobacteria, Beta proteobacteria, Gammaproteobacteria, and Bacteroidetes. The AAT-domain sequences from the MocRs of the six phyla were merged into a single dataset, which was filtered at 50% sequence identity level by the cd-hit tool. The final taxonomical composition of the AAT-domain set, accounting for a total of 1331 sequences, is reported in Table 1. Sequences of MocRs described in the literature (Table 2) have been added to the final multiple sequence alignment.

### Multidimensional scaling analysis:

**Figure 1:**
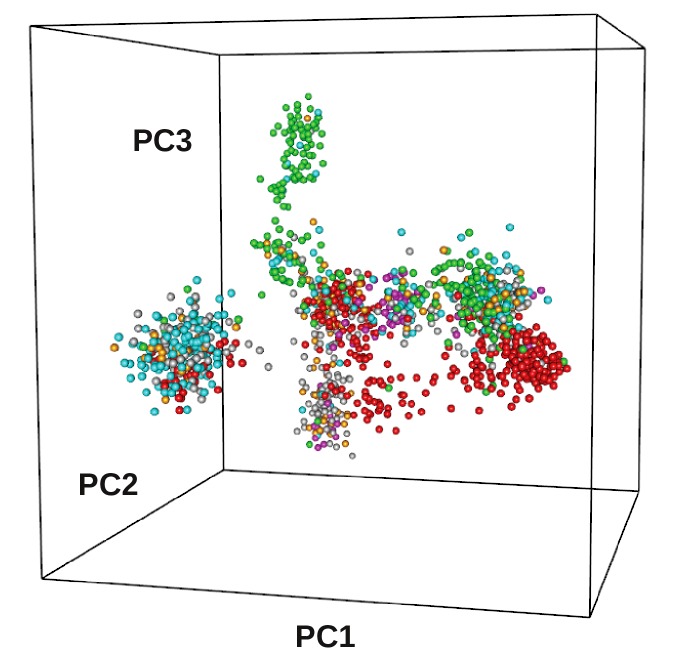
Three-dimensional representation of sequence space obtained with the multidimensional scale analysis implemented in the R package "bios2mds" of the aligned AAT-like domain. 3D space is defined by the first three components of the MDS (PC1, PC2, PC3). Distances were based on the pairwise BLOSUM30 alignment score. Colours indicate phylum: Actinobacteria green dot; Firmicutes red dot; Alphaproteobacteria cyan dot; Betaproteobacteria orange dot; Gammaproteobacteria blue dot; Bacteroidetes grey dot

To test for the existence of AAT-domain subgroups within the collected MocR set, the multidimensional scaling analysis (MDS) implemented in the R package "bios2msd" [43] has been applied. The distance matrix containing the differences calculated between all possible pairs of sequences within the AAT-domain multiple sequence alignment, has been calculated using the BLOSUM30 matrix. Figure 1 reports the projection of sequence distances onto the 3D space defined by the first three components of the MDS analysis. Distribution in the 3D space suggests the presence of at least three groups each including sequences from different phyla.

### Cluster analysis and HMM profile search:

**Figure 2:**
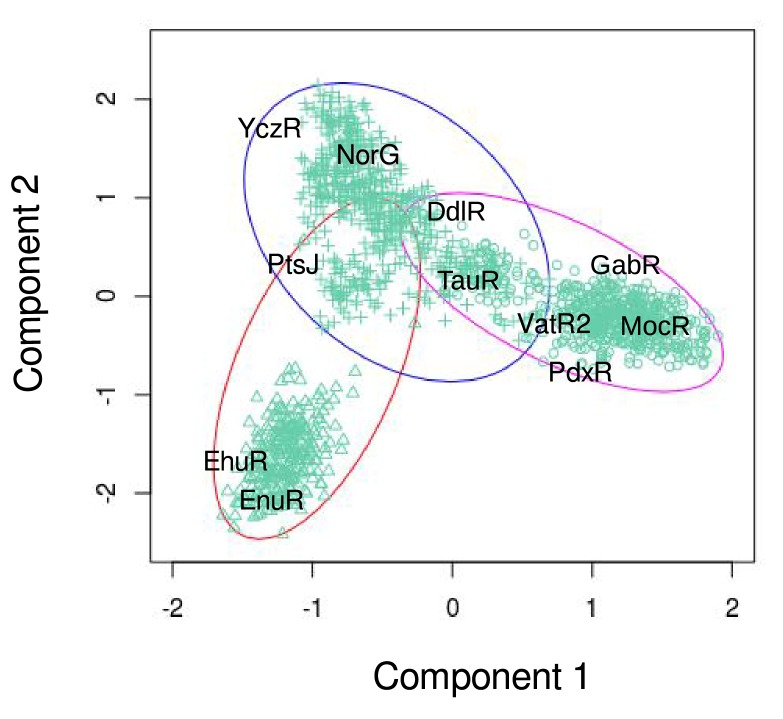
Bivariate plot visualizing the clustering of the AAT-domain sequences. Clusters are delimited by ellipses and the corresponding members denoted by different symbols. Labels denote the positions of the reference MocRs.

The R "Kmeans" function has been applied to the distance matrix of the AAT-like domains. To establish the optimal number of clusters, silhouette score analysis [47] has been applied as available in the "bios2mds" package. Briefly, silhouette score measures how well data points are classified when assigned to a set of clusters. The measure takes into account the tightness of the clusters and the separation between them. The silhouette score values range from 1.0 to 1.0 that indicates very poor or optimal classification, respectively. Silhouette score can be calculated assuming different numbers of clusters. The cluster number showing the highest score suggests the best clustering. In this case, the highest score (0.60) was obtained assuming 3 clusters. The same procedure applied to the randomized 1331 sequences had a peak silhouette score of only 0.23 for 4 clusters (results not shown). Figure 2 reports the projection of the three clusters onto the space defined by the first two components. The position of the eleven reference MocRs is denoted by labels. Each cluster has been named after one of the enclosed MocRs: cluster GabR, PtsJ, and EnuR (Table 3). Phylum composition of the three clusters is reported in Table 3. Except for Bacteroidetes, it appears rather homogeneous.

AAT-domain sequences assigned by K-mean clustering to each of the three clusters have been separately aligned with clustal omega. A Profile Hidden Markov Model (HMM) has been calculated for each multiple alignment with the program HMMbuild. Each HMM profile has been utilized as a HMM search query over the Pfam-A databank. Only hits showing an E-value lower than 0.1 were considered. The distribution of the Pfam hits obtained by each HMM profile is displayed in Table 4. The distribution clearly pinpoints that the three AAT-domain clusters show variable degrees of similarity to different families of fold type-I PLP proteins.

### Sequence and logo comparison:

**Figure 3:**
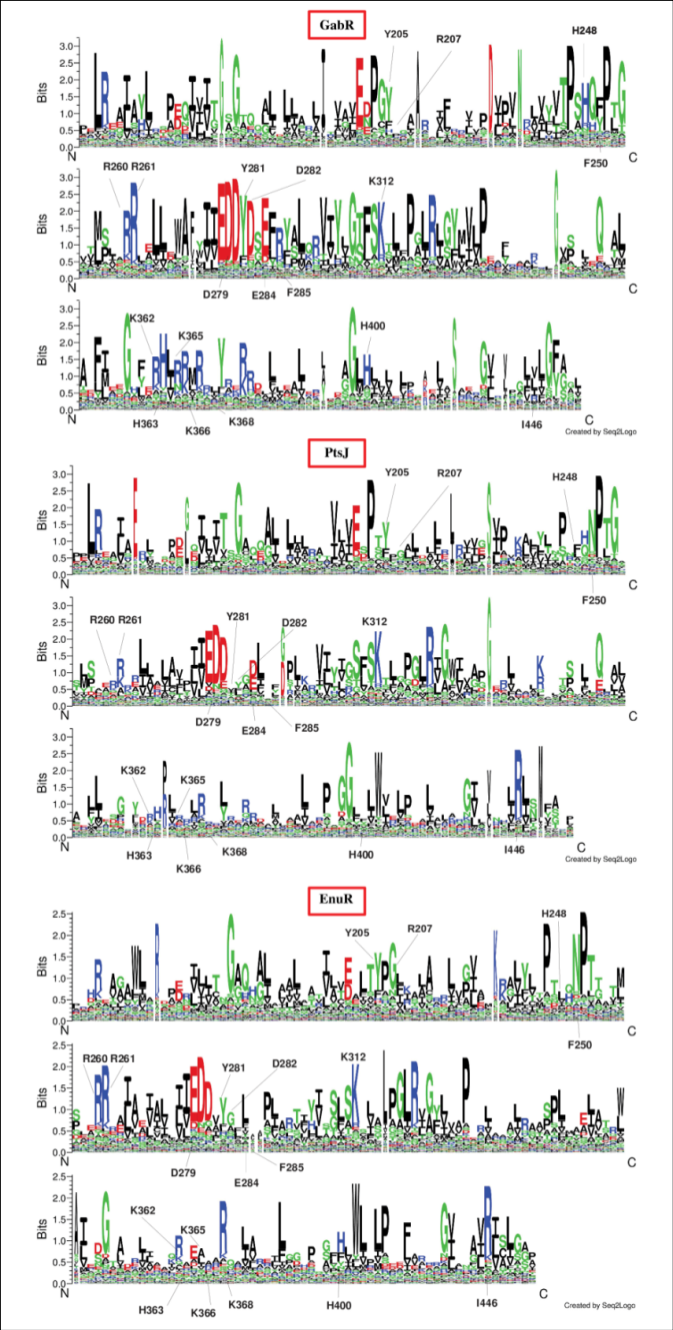
Logos comparison calculated through the Web site "Seq2Logo" for the block portions of GabR, PtsJ, and EnuR clusters (indicated by red boxes). Residues are represented by their one-letter code and coloured according to chemical properties. Labels indicate the sites discussed in the text according to the GabR 5x03 sequence numbering framework.

**Figure 4:**
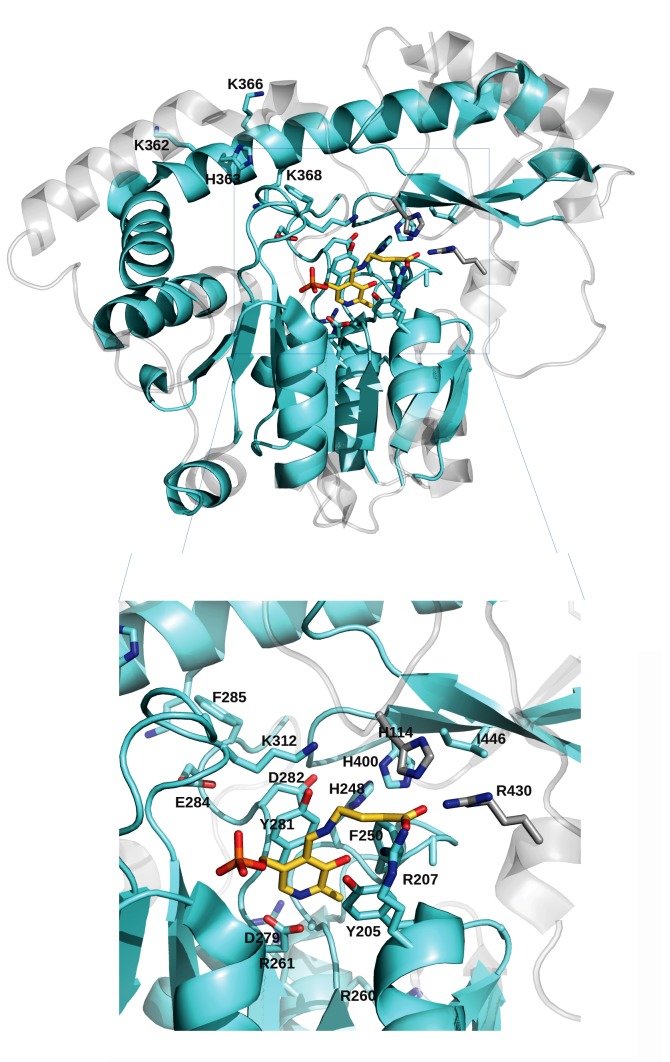
: Three-dimensional structure of GabR from Bacillus subtilis in complex with PLP and γ-aminobutyric acid (PDB code 5x03). Transparent grey ribbon depicts the entire 5x03 monomer while the cyan segments denote the portions corresponding to the AAT-domain blocks. Lower panel displays a detail of the active site region where residues discussed in text are represented by stick models and labelled according to 5x03 numbering system. Cyan and grey stick residues mark the positions differing in the three MocR clusters and those discussed in the text, respectively. Yellow stick model represents the PLP bound to γ-aminobutyrate.

To spot the sequence features discriminating the three clusters, corresponding Logos have been compared. To avoid noise due to inaccuracies in sequence alignment, Logo comparison has been restricted to blocks, namely to portions of the multiple sequence alignment made of at least three consecutive columns each containing not more than 90% gaps (Figure 3). The position of the blocks have been projected onto the three-dimensional structure of the AAT-domain of GabR from Bacillus subtilis (corresponding to the PDB file 5x03) and shown in Figure 4. The selected blocks cover the PLP-binding site and part of the surrounding areas including the α-helix connecting the major and minor domains and a twostrand β-sheet therein. The positions characterizing the MocR AATdomain clusters have been identified and listed in Table 5. Structural and functional role has been associated to these positions by mapping onto the GabR reference structure 5x03. In addition to the variant residues pinpointed by Logo comparison, particular attention has been paid to the residues that in 5x03 are involved in effector and cofactor interactions (Table 5). Interestingly, the residue interacting with the γ-aminobutyrate ligand in 5x03 is localized in sequence portions generally not conserved across the three MocR clusters, hit by insertions/deletions during evolution.

## Discussion

The MocR regulators are chimeric proteins emerged from ancestral fusion events between a gene coding for an HTH domain and an effector/dimerization domain belonging to a vast and diversified enzyme superfamily, the fold type-I PLP-dependent enzymes [48]. Aspartate aminotransferase (AAT) is archetypical for the superfamily often denoted as AAT-like [19]. Gene fusion is one of the main mechanisms driving the molecular evolution of proteins along with gene duplication, fission, recombination and loss of fragments [49]. HTH and AAT-domains are coded by genes of very ancient origin and have been exposed to a long and extensive molecular evolution, which led to a massive functional diversification. Typically, sequence similarity among members of different fold type-I families can be extremely low (as low as a few percentage identity) although three-dimensional structure is considerably well conserved [15,50]. Somehow unexpectedly, the molecular evolution of the AAT-like superfamily has branched out into a family of bacterial transcriptional regulators that lost, as far as it is know today, enzymatic activity while maintaining the ability to bind PLP and specific effectors [27,28]. Starting from this picture, it has been tested whether the vast MocR family can be subdivided into subgroups possibly emerged after a single, ancestral fusion event between prototypic HTH and AAT-like genes or after different, independent events involving already catalytically specialized AAT-like ancestral enzymes.

The results obtained by MDS and clustering analyses support the notion that at least three MocR subgroups can be distinguished. The clustering does not reflect apparently the taxonomic classification of the source species since MocR sequences belonging to the same bacterial phylum agglomerate into different clusters. Consequently, clustering seems to reflect functional rather than evolutionary proximity. Cluster analysis attributes the reference MocRs (Table 3) to three subgroups: GabR subgroup contains the MocRs involved in the regulation of expression of genes with enzymatic activity; PtsJ subgroup contains MocRs involved in the regulations of expression of PLP-dependent enzymatic activity and/or membrane transporters such as NorG (or putatively YczR); finally, the least populated EnuR subgroup collects the MocRs connected to ectoine metabolism.

The attempt to identify the positions characterizing the MocR sequences assigned to different clusters on the basis of the multiple sequence alignment was compounded by the high dissimilarity of the sequences: the final alignment contains many long insertions/deletions and the average pairwise percentage identity was around 20%. For this reason, attention has been focussed onto conserved blocks. Interestingly, the blocks add up to a structural "core" surrounding the PLP binding site of the GabR AAT-domain structure along with the helix connecting the large and small domain of the single subunit (Figure 4). This core may represent the minimal set of structural elements necessary to a functional MocR fold able to bind PLP. Indeed, Asp279 (interacting with pyridine nitrogen) and Lys312 (forming the Schiff-base with the PLP cofactor) are conserved across all clusters (Figure 3 and Figure 4). Among the residues, noteworthy are those in contact with the PLP cofactor, namely (according to the GabR numbering system) Phe250 and Tyr281 (Figure 3 and Figure 4). GabR Phe250 is in contact with the phenolate side of the PLP ring and is conserved in the GabR cluster while it is replaced by the hydrophilic residue Asn in the other two clusters (Table 5). Tyr281 seems to be typical of GabR cluster; in the other clusters the position is frequently occupied by Ala, Val or Ile. PLP stacking Tyr205 is conserved (Table 4) and Phe replaces it frequently. The aromaticity of the positions 205 thus seems to be a requirement for a functional MocR whiles the "aromatic triplet" [51] formed by Tyr205, Phe250 and Tyr281 is distinctive of theGabR subgroup. Other residues of the “second shell” surrounding the PLP binding site differ in the three clusters (Figure 4 and Table 5). Overall, the structural environment in which PLP pyridine ring is immersed varies in the three clusters, suggesting diversity of effector specificity. Residues interacting with the GABA carboxylate in GabR are: His114, Arg207 and Arg430 (Figure 4). His114 and Arg430 do not occur within conserved alignment blocks. Arg207 occurs within a block; the alignment suggests that the position is rather variable in the three clusters thus pointing again to a possible role in determining effector specificity (Figure 4 and Table 5).

HMMsearch suggests that the three clusters have variable degrees of similarity to the Pfam families corresponding to different fold type-I families. All MocR profiles displays high similarity to the Pfam Aminotran_1_2 family that collects most of the fold type-I PLP dependent aminotransferases and to the family Aminotran_MocR, collecting the major domain of triptofanases. However, GabR cluster profile retrieves very few sequences belonging to other fold type-I families while PtsJ and EnuR profiles do (Table 4). A sharp discrimination between clusters PtsJ and EnuR cannot be drawn. However PtsJ profile retrieves more Cysteine desulfurase (Pfam code: PF00266) and Ornithine transaminase-like (PF00202) sequences than the EnuR profile. At variance with the former, the latter profile retrieves more Ornithine decarboxylase-like sequences (PF01276). It is interesting to mention that YczR, putative regulator of expression of membrane protein involved in sulfur compounds transportation, belongs to the PtsJ subgroup that display more affinity to the Cysteine desulfurase-like Pfam family.

## Conclusion

The results reported here support the hypothesis that the MocR regulators emerged after independent ancestral fusion events between a HTH domain and at least three already catalytically specialized PLP dependent enzymes of fold type-I. This hypothesis is also coherent with the conception that regulation machinery should emerge after evolution of the metabolic pathway under its control as, for example, in the case of prokaryotic BdzR regulator involved in the anaerobic degradation of benzoate [52]. However, the possible contribution of lateral gene transfer to the observed MocR distribution cannot be neglected because of its relevant role in bacterial evolution [53,54]. The story of MocR regulators is intertwined to the complex evolution process that led to the catalytically versatility of AAT-like enzymes. The same versatility must be reflected in the yet unexplored functional heterogeneity of MocR population. The classification presented here can assist in the study of new MocRs and can support rational design of experiment for functional characterization.

## Conflict of Interest

none

## Figures and Tables

**Table 1:** Distribution of the taxonomic origin of AAT-like domain sequences at 50% pairwise sequence identity level

Phylum	counts
Actinobacteria	248
Alpha proteobacteria	260
Bacteroidetes	57
Beta proteobacteria	143
Firmicutes	369
Gamma proteobacteria	254

**Table 2:** Reference MocRs

Databank accession number	Label^a^	Function^b^	Source organism
P49309	MocR	Probable rhizopine catabolism regulatory protein	Rhizobium meliloti
D5AKX9	TauR	Transcriptional activator, which is essential for taurine-dependent expression of the tpa-tauR-xsc operon	Rhodobacter capsulatus
P94426	GabR	Activates the transcription of the gabTD operon.	Bacillus subtilis
Q8NS92	PdxR	regulatory function in pyridoxine biosynthesis	Corynebacterium glutamicum
Q2YUS3	NorG	Positively regulates the expression of the NorB efflux pump and negatively regulates the expression of the AbcA efflux pump	Staphylococcus aureus
P40193	PtsJ	Transcriptional repressor of the pdxK gene	Salmonella typhimurium
C0ZDG2	DdlR	Transcriptional regulator of D-alanyl-D-alanine ligase	Brevibacillus brevis
A6T5K2|	YczR	Putative transcriptional regulator of the expression of YczE genes	Klebsiella pneumoniae
WP_104279468	VatR2	Regulates expression of virulence factors, membrane and secreted proteins, and signal transducing proteins	Clavibacter michiganensis
YP_002824530	EhuR	Negative regulation of Ectoine uptake and catabolism	Sinorhizobium meliloti
AMR55826	EnuR	Transcriptional regulation of ectoine catabolism	Ruegeria pomeroyi

**Table 3:** Taxonomic composition of the three subgroups as resulting from K-means clustering

Cluster name	Sequence counts	Reference MocRs^a^	Actinobacteria	Alpha^b^	Bacteroidetes	Beta^c^	Gamma^d^	Firmicutes
GabR	535	GabR	106	99	22	45	76	187
PdxR
TauR
MocR
VatR2
PtsJ	555	PtsJ	132	45	34	76	123	145
NorG
DdlR
YczR
EnuR	242	EnuR	10	116	1	23	55	37
EhuR

**Table 4:** Pfam family hits retrieved by the Profile HMM calculated for each cluster, after a HMMsearch over the Pfam-A databank

Pfam hits^c^
Pfam codes^a^	GabR^b^	PtsJ^b^	EnuR^b^
PF00155.21 Aminotran_1_2 (Aminotransferase class I and II)	98.47	78.96	82.81
PF12897.7 Aminotran_MocR (Alanine-glyoxylate aminotransferase)	1.43	0.98	1.07
PF01053.20 Cys_Met_Meta_PP (Cys/Met metabolism PLP-dependent enzyme family)	0	2.77	2.54
PF00266.19 Aminotran_5 (Aminotranferase class V)	0.09	5.64	2.9
PF00202.21 Aminotran_3 (Aminotransferase class III)	0	2.9	1.53
PF01041.17 DegT_DnrJ_EryC1 (DegT/DnrJ/EryC1/StrS aminotransferase family)	0.02	7.37	6.35
PF01276.20 OKR_DC_1 (Group III pyridoxal-dependent decarboxylases)	0	0.8	2.48
PF01212.21 Beta_elim_lyase (Beta-eliminating lyase)	0	0.59	0.31

**Table 5:** Sequence sites differing among clusters

Position^a^	GabR^b^	GabR^c^	PtsJ^c^	EnuR^c^	Function^d^
205	Tyr	Tyr/Phe	Tyr/Phe	Tyr/Phe	Stacking with PLP pyridine ring
207	Arg	Gly	Gly/Asn	Gly/Asn	Salt-bridge to GABA carboxylate
248	His	His/Arg	Hphobic	Hphobic	Interaction with His400
250	Phe	Phe/Tyr	Asn	Asn	Interaction with PLP
260	Arg	Arg	Variable	Arg	First turn of helix 257-271
261	Arg	Arg	Variable	Arg	'
281	Tyr	Tyr	Val/Ile	Val/Ile	Stacking with PLP
282	Asp	Asp	Hphobic	Tyr	Interaction with position 248 and 400
284	Glu	Asp/Glu	Asp/Glu	Hphobic	Interact with Tyr360
285	Phe	Phe	Ile	Ile	Interact with Glu284
362	Lys	Lys	Variable	Variable	Basic surface patch on the helix connecting the two domains
363	His	His	Variable	Hphobic	"
365	Lys	Lys/Arg	Variable	Variable	"
366	Lys	Lys/Arg	Variable	Variable	"
368	Lys	Lys/Arg	Arg/Variable	Arg/variable	"
400	His	His	Variable	His	Interaction with Asp282
446	Ile	Hphobic	Arg	Arg	Interaction with His400
